# Effect of Predatory Bacteria on Human Cell Lines

**DOI:** 10.1371/journal.pone.0161242

**Published:** 2016-08-31

**Authors:** Shilpi Gupta, Chi Tang, Michael Tran, Daniel E. Kadouri

**Affiliations:** 1 Department of Oral Biology, Rutgers School of Dental Medicine, Newark, NJ, United States of America; 2 Department of Medicine and the Center for Emerging Pathogens, Rutgers, New Jersey Medical School, Newark, NJ, United States of America; Institut Pasteur Paris, FRANCE

## Abstract

Predatory bacteria are Gram-negative bacteria that prey on other Gram-negative bacteria and have been considered as potential therapeutic agents against multi-drug resistant pathogens. *In vivo* animal models have demonstrated that predatory bacteria are non-toxic and non-immunogenic in rodents. In order to consider the use of predatory bacteria as live antibiotics, it is important to investigate their effect on human cells. The aim of this study was to determine the effect of *Bdellovibrio bacteriovorus* strains 109J and HD100, and *Micavibrio aeruginosavorus* strain ARL-13 on cell viability and inflammatory responses of five human cell lines, representative of clinically relevant tissues. We found that the predators were not cytotoxic to any of the human cell lines tested. Microscopic imaging showed no signs of cell detachment, as compared to predator-free cells. In comparison to an *E*. *coli* control, exposure to higher concentrations of the predators did not trigger a significant elevation of pro-inflammatory cytokines in four of the five human cell lines tested. Our work underlines the non-pathogenic attributes of predatory bacteria on human cells and highlights their potential use as live antibiotics against human pathogens.

## Introduction

Traditional antimicrobial agents are increasingly becoming ineffective as the number of multi-drug resistant (MDR) pathogens increase. A drastic decline in the rate of development of new antibiotics is fueling this global health issue, driving researchers to search for novel therapies against infections caused by these MDR pathogens [1]. One such group of potential therapeutic agents is predatory bacteria [2]. *Bdellovibrio bacteriovorus*, a delta-proteobacterium, first isolated from soil in 1963 [3, 4] and *Micavibrio aeruginosavorus*, an alpha-proteobacterium, first isolated from wastewater in 1983 [5, 6], are both obligate predators. These are Gram-negative bacteria that prey on other bacteria using different strategies. *B*. *bacteriovorus* are periplasmic invaders that enter the prey and use its cellular content to replicate, ultimately lysing the cell and moving on to the next prey cell [7]. In contrast, *M*. *aeruginosavorus* feed externally without penetrating the prey cell as they “leech” to their prey and divide by binary fission [5, 8].

In recent years, the predatory ability of *B*. *bacteriovorus* and *M*. *aeruginosavorus* is increasingly drawing more interest as potential therapy against Gram-negative human pathogens, especially those highly resistant to conventional antibiotic treatments. In previous studies, the predatory bacteria were found to be able to attack MDR Gram-negative bacteria, thereby proving useful where other antimicrobials fail [9]. These potential biological control agents have been shown to rapidly reduce Gram-negative bacteria grown planktonicly in suspended cultures as well as surface attached biofilms [10, 11]. As for any new therapeutic, it is essential to understand the potential risks associated with the use of predatory bacteria as a live antibiotic. Work conducted in chicken and mice models have already proven that predatory bacteria might be non-toxic and non-immunogenic. A study conducted by Sockett *et al*. found that *B*. *bacteriovorus* significantly reduced the number of *Salmonella* in infected live-chicks compared to the untreated controls, without having any adverse effect on their wellbeing [12]. In a more recent report, no reduction in viability of mice was reported following introduction of *B*. *bacteriovorus* and *M*. *aeruginosavorus* via the lung and tail vein [13]. In addition, the study found that the predatory bacteria did not produce any sustained immune response and were efficiently cleared from the inoculated organs [13]. Although using animal models to examine the effect of predatory bacteria *in vivo* is essential, these models provide only a partial understanding of any adverse effects that might occur while introducing the predators to human subjects in order to treat an infection.

A first step in understanding the effect of predatory bacteria in the human body is to examine its impact on human cell lines. In a previous study, the non-toxic effect of *B*. *bacteriovorus* and *M*. *aeruginosavorus* was successfully demonstrated using human corneal-limbal epithelial cells as an *in vitro* model of ocular tissue [14]. In the current study, we aimed to broaden our understanding regarding the impact of predatory bacteria on human cells. 109J and HD100 strains of *B*. *bacteriovorus* and ARL-13 strain of *M*. *aeruginosavorus* were chosen for this study as they have previously shown to prey on a range of human pathogens [2]. Five human cell lines, representative of different tissues, were challenged with high doses of these predatory bacteria and the change in cell viability and inflammatory response was measured. Our data demonstrated that the predators were not cytotoxic to the human cells and did not trigger an elevated inflammatory response. Our results add to the existing published findings that underline the non-pathogenic attributes of predatory bacteria and highlight their potential to be used as live antibiotics as an adjunctive or alternative to traditional antibiotics.

## Materials and Methods

### Bacterial strains and growth conditions

The predatory bacteria used in the study were *Bdellovibrio bacteriovorus* strains 109J (ATCC^®^ 43826^™^) and HD100 (ATCC^®^ 15356^™^) [15], and *Micavibrio aeruginosavorus* strain ARL-13 [8]. Predators were cultured as described previously [2]. *Escherichia coli* WM3064, a diaminopimelic acid (DAP) auxotroph, was used as prey and grown overnight in LB medium supplemented with 0.3 mM DAP [16, 17]. Predator stock lysates were prepared by co-culturing the predatory bacteria with prey in HEPES buffer (25 mM) supplemented with 3 mM MgCl_2_ and 2 mM CaCl_2_ [18]. The co-cultures were incubated on a rotary shaker at 30°C for 24 and 72 hours for *B*. *bacteriovorus* and *M*. *aeruginosavorus* respectively, until cultures appeared clear. To obtain higher concentration of predators for the experiments, fresh cultures were prepared from the stock lysates as described previously [13] with some modifications. For *Bdellovibrio* cultures, 10 ml of washed overnight cultures of *E*. *coli* WM3064 (~1 × 10^9^ CFU/ml) and 10 ml of stock lysates were added to 80 ml of HEPES medium and incubated for 24 hours. For *Micavibrio*, 25 ml of *Micavibrio* stock lysates and 25 ml of *E*. *coli* WM3064 added to 200 ml of HEPES medium and incubated for 72 hours.

For predator concentration and purification, the above co-cultures were passed twice through 0.45-μm Millex^®^-HV pore-size filter (Millipore, Billerica, MA, USA) to remove any residual prey cells or cell debris (filtered lysate). The filtered lysates were further washed from residual cell debris and concentrated by three sequential centrifugations at 29,000 ×g for 45 minutes at 10°C using a Sorvall LYNX 4000 centrifuge (Thermo Fisher Scientific Inc). Each time the pellets were suspended in 50 ml of phosphate buffered saline (PBS). The final pellets were re-suspended in 1–2 ml PBS buffer to reach an optical density (OD_600_) of 0.2±0.02 for *Bdellovibrio* and 0.1±0.02 for *Micavibrio* which corresponded to a plaque-forming unit (PFU) value of between ~5x10^9^ to 5x10^10^ /ml and ~5x10^8^ to 5x10^9^ /ml, respectively. PFU enumeration was done to determine predator concentrations using the standard double-layer agar method [13, 19]. Since the epibiotic predator *M*. *aeruginosavorus* has relatively limited growth capabilities in comparison to the periplasmic *B*. *bacteriovorus*, the concentration used for *M*. *aeruginosavorus* was lower than that of *B*. *bacteriovorus*, as it was technically challenging to obtain consistently higher than 10^9^ PFU/ml cells. To ensure that the predator samples were free from prey cells and contamination, aliquots were plated on DAP (0.3 mM) supplemented LB agar, Nutrient agar, and TSB blood agar plates.

*P*.*aeruginosa* strain PA14 [14] and *E*.*coli* ATCC strain 43888 (serotype O157:H7) [20, 21] were used as positive controls for cytotoxicity and cytokine assays, respectively. Cultures were grown overnight in LB medium at 37°C.

### Cell cultures

Five human cell lines were used in this study. These included the adherent cell lines—human keratinocytes (HaCaT) [22], human liver epithelial cells (HepG2) (ATCC^®^ HB-8065^™^), and human kidney epithelial cells (HK-2) (ATCC^®^CRL-2190^™^); loosely adherent human spleen monocytes (MD) (ATCC^®^ CRL-9850^™^); and suspension human blood monocytes (THP-1) (ATCC^®^ TIB-202^™^). HaCaT cell line was kindly provided by Dr. Zurawski at Walter Reed Army Institute of Research and maintained in Dulbecco’s Modified Eagle Medium supplemented with high glucose, GlutaMAX^™^, sodium pyruvate, and 10% Fetal Bovine Serum (FBS), (reagents obtained from GIBCO, Life Technologies^™^). All the other cell lines were obtained from American Type Culture Collection (ATCC, Manassas, Virginia) and maintained according to ATCC prescribed guidelines. Cell culture media and supplements were obtained from Sigma-Aldrich, St. Louis, MO. HepG2 and MD cell lines were cultured in Eagle’s minimum essential medium and Iscove’s modified Dulbecco’s medium with L-glutamine, respectively, both supplemented with 10% FBS. In addition to base medium, MD cells were supplemented with 2-mercaptoethanol (0.05 mM), hypoxanthine (0.1 mM), thymidine (0.016 mM) and 10% FBS. HK-2 cells were maintained in Keratinocyte serum free medium supplemented with bovine pituitary extract (0.05 mg/ml) and human recombinant epidermal growth factor (5 ng/ml). Human blood monocytes (THP-1) were cultured in RPMI 1640 medium supplemented with 2-mercaptoethanol and 10% FBS. THP-1 cells were differentiated into macrophages using 100 nM PMA (Phorbol 12-myristate 13-acetate) and activated with interferon-γ (500 IU/ml) [23]. Cell lines were seeded without antibiotics to prevent interference in subsequent assays. The cell cultures were maintained at 37°C with 5% CO_2_ in a standard bench-top CO_2_ incubator (VWR, Radnor, PA).

### Cytotoxicity assays

To examine the effect of predatory bacteria on human cells, the cell lines were challenged with the three predators. Changes in cell viability were measured using PrestoBlue^®^ Cell Viability Reagent (Invitrogen, Carlsbad, CA). Metabolically active human cells quickly reduced the reagent, providing a quantitative measure of viability and cytotoxicity. Predators were prepared as described above. The adherent human cells were seeded into 24-well flat bottom cell culture plates (Corning^®^ Costar^®^ plates from Sigma-Aldrich, St. Louis, MO) at a density of 5x10^4^ cells/well in 500 μl culture media. The plates were incubated for 24 hours at 37°C with 5% CO_2_ to reach a confluence of 80–90%. HepG2 cells were seeded at a lower density of 1x 10^4^ cells/well and harvested at 60–70% confluence, as these cells tend to form loosely adherent 3D structures at higher densities [24]. THP-1 cells were seeded at a higher density of 5 x 10^5^ cells/well and treated with 1 μl/ml each of PMA and IFN- γ for 48 hours prior to the experiment in order to induce macrophage differentiation and cell adherence [23].

After obtaining the required cell confluence, growth medium was removed and substituted with 450 μl/well fresh antibiotic free media and 50 μl/well of purified predator bacteria (~1.0 x 10^9^ PFU/well for *B*. *bacteriovorus* strains HD100 and 109J; ~1 x 10^8^ PFU/well for *M*. *aeruginosavorus* ARL-13). Predator-free PBS was used as a negative control while 50 μl of PBS washed overnight culture of *P*. *aeruginosa* PA14 (~1 x 10^8^ CFU/well) and Triton X-100 (0.5%) were used as positive controls for cytotoxicity [14]. Cell cultures were incubated at 37°C with 5% CO_2_ for 24 hours, after which the wells were washed three times with a solution containing PBS and the antibiotics, hygromycin (500 μg/ml) and gentamicin (250 μg/ml), to eliminate any residual bacteria. Thereafter, 540 μl of a solution containing fresh media and the antibiotics was added to each well followed by 60 μl/well of PrestoBlue^®^ reagent. The plates were incubated for 30 minutes and the fluorescence was measured at 560 nm (excitation) and 590 nm (emission) using a BioTek^™^ Synergy H1 Multi-Mode Reader.

For loosely adherent human spleen monocytes (MD), the cells were treated with the predators and controls in the 24-well culture plate as described above. Following 24-hour incubation with the various treatments, the MD cells were removed gently from the plate using a fixed blade cell scraper (CytoOne, USA Scientific, FL). The cells were transferred to a 2 ml micro centrifuge tube and residual bacteria were removed by centrifugation at 240 ×g for 10 minutes. After the washing step, the cells were placed back in a 24-well cell culture plate and PrestoBlue^®^ reagent was added and analyzed as described before. Each experiment was conducted at least twice in quadruplicate (4 cell culture wells per sample) for each cell line.

Data were analyzed, calculated and presented as ‘percent survival’ using the following equation: [1- (negative control value—experimental value) / (negative control value—positive control value)] x 100. To standardize percent viability, fluorescence measured from the predator-free PBS sample (negative control) was calculated as 100% survival and that from Triton-X sample (positive control) was calculated to be 0% survival.

### Cell viability imaging

To visualize cell integrity and viability, cells were grown and treated as described above, followed by addition of Calcein AM^™^ (Thermo Fisher, Waltham, MA) rather than PrestoBlue^®^. Calcein AM^™^ (CaAM) is a cell-permeant dye that can be used to determine cell viability by fluorescence imaging [25]. Sixty μl of CaAM/PBS working solution (2 μM CaAM) was added to 540 μl of fresh serum-free media containing antibiotics and added to each well of the 24-well plate. After incubation for 30 minutes at 37°C, fluorescent and phase contrast images were captured using an EVOS FL digital inverted fluorescence microscope (Thermo Fisher, Waltham, MA) equipped with an adjustable intensity LED light source. Experiments were conducted twice in quadruplicate for each cell line.

### Cytokine assays

To measure the effect of predatory bacteria on cell cytokine profile of the exposed human cell lines, samples were analyzed for the following cytokines: GM-CSF, IFN-γ, IL-10, IL-12p70, IL-1β, IL-2, IL-6, IL-8 and TNF-α. To this end, predator samples were prepared as described above. The cell cultures were grown as described for the cell viability assays. After reaching ~90% confluence, the media was removed from the wells and substituted with 225 μl of fresh media and 25 μl each of predator bacteria and controls. For this experiment, predator-free PBS served as the negative control and *E*. *coli* ATCC 43888 (~10^8^ CFU/ml) was used as the positive control. Preliminary observations had found this strain of *E*. *coli* to have a reduced lytic effect on the cells, as compared to the *P*. *aeruginosa* strain PA14, allowing the cells to survive for the duration of the experiment.

Following 4- and 24- hours of incubation, 250 μl of supernatant was collected from each well. To remove any remaining cells, samples were centrifuged for two minutes at 2000 ×g in a micro centrifuge (Eppendorf, NY). The supernatants were placed in fresh micro-centrifuge tubes and sent to Molecular Resource Facility at Rutgers New Jersey Medical School for cytokine analysis. Cytokine levels were quantified using Milliplex^®^ MAP cytokine/chemokine magnetic bead based immunoassay (HCYTOMAG-60K, EMD Millipore, MA) according to manufacturer’s guidelines with the help of MAGPIX^®^ instrument powered by xMAP^®^ technology (Luminex). Data were analyzed using Milliplex^®^ Analyst 5.1 software. Data were presented as fold-change induction of cytokines in relation to PBS negative control using the following equation: [(Mean experimental value—Mean negative control value) / Mean negative control value]. Experiments were conducted twice in quadruplicate for each cell line.

### Statistical analysis

Graphpad Prism 6 software was used to perform One-way ANOVA followed by Tukey’s multiple comparison test.

## Results

### Cytotoxicity assays

**Fig 1:**
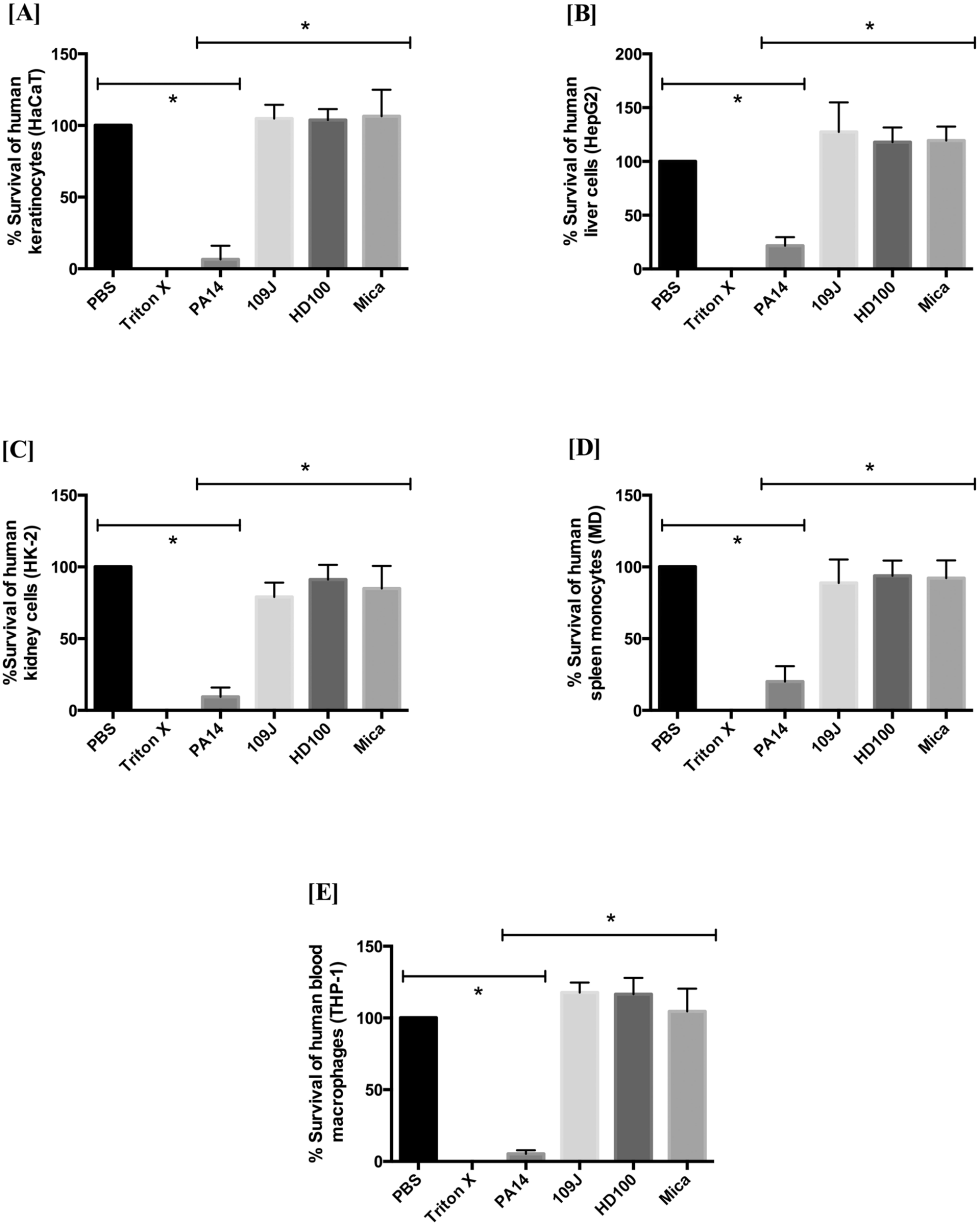
Cytotoxicity analysis of human cells following 24 hours exposure to predatory bacteria. PrestoBlue^®^ cell viability reagent was used to measure changes in cell viability of [A] human keratinocytes (HaCaT), [B] human liver cells (HepG2), [C] human kidney cells (HK-2), [D] human spleen monocytes (MD), and [E] human blood macrophages (THP-1), following a 24 hour exposure to *B*. *bacteriovorus* strains 109J, HD100 (~1 x 10^10^ PFU/ml) and *M*. *aeruginosavorus* strain ARL-13 (~1 x 10^9^ PFU/ml). PBS was used as a negative control, and Triton X-100 (0.5%) and *P*. *aeruginosa* strain PA14 (~1 x 10^9^ CFU/ml) as positive controls. Experiments were conducted twice in quadruplicate for each sample. Asterisks indicate significant differences (p<0.0001, ANOVA with Tukey’s test) between negative and positive controls, and between the experimental samples and positive controls. Error bars indicate one standard deviation.

When considering the use of predatory bacteria as a live therapeutic agent against human pathogens, it is important to determine whether these bacteria have a cytotoxic effect on human tissues. To this end, cell viability of the five human cell lines was measured after they were exposed to *B*. *bacteriovorus* and *M*. *aeruginosavorus* for 24 hours. PrestoBlue^™^ viability reagent was added to the cells after washing them with media containing antibiotics to prevent further growth of bacteria not removed by washing. As expected, *P*. *aeruginosa* strain PA14 was found to be cytotoxic to all the cell lines, decreasing the viability significantly (p<0.0001) by 80–95% as compared to the PBS control. For all cells lines tested, viability in the cells exposed to the predators was found to be significantly higher (p<0.0001) than that exposed to *P*. *aeruginosa* (Fig 1).

As seen in Fig 1A, *P*. *aeruginosa* significantly reduced the viability of HaCaT cells (human keratinocytes) by 93.5%, while exposure to the predators increased cell viability by 4–7% (p>0.05), compared to the PBS control. For liver (HepG2) and kidney (HK-2) epithelial cells, *P*. *aeruginosa* reduced the cell viability by 78.5% and 90.5%, respectively, while treatment with predators increased the viability of HepG2 cells by 18–25% (p>0.05). A non-significant reduction of 21% (p>0.05) was noted in viability of HK-2 cells exposed to *B*. *bacteriovorus* 109J (Fig 1B and 1C). We found that the cell viability was reduced by 80% and 94.8% in *P*. *aeruginosa*-exposed spleen monocytes (MD cells) and blood macrophages (THP-1 cells) respectively. Treatment with predators decreased the viability of MD cells by 7–12% (p>0.05), while there was an increase of 5–17% (p>0.05) in viability of THP-1 cells (Fig 1D and 1E). Hence, incubation with predatory bacteria did not cause any significant reduction (p>0.05) in the human cell viability when compared to the cells exposed to predator-free PBS, thereby suggesting that these bacteria are non-toxic to the human cells.

In order to validate that predatory bacteria can survive in the cell culture media, the predators and *P*. *aeruginosa* PA14 were incubated with each of the complete cell culture medium (HaCaT, HepG2, HK-2, MD, THP-1) at 37°C with 5% CO_2_ for 24 hours and viability counts were done using PFU and CFU enumeration. PBS was used as a control. Limited reductions in predator cell viability of < 1 log_10_ was noted with all the cell culture media. PFU counts for *B*. *bacteriovorus* 109J and HD100 showed 0.92 and 0.60 log_10_ reductions, respectively, in the presence of HaCaT cell medium. Incubation in HepG2 medium reduced the *M*. *aeruginosavorus* population by 0.98 log_10_. *P*. *aeruginosa* PA14 counts showed an increase of 0.65 log_10_ when incubated with THP-1 cell medium.

### Cell viability imaging

**Fig 2:**
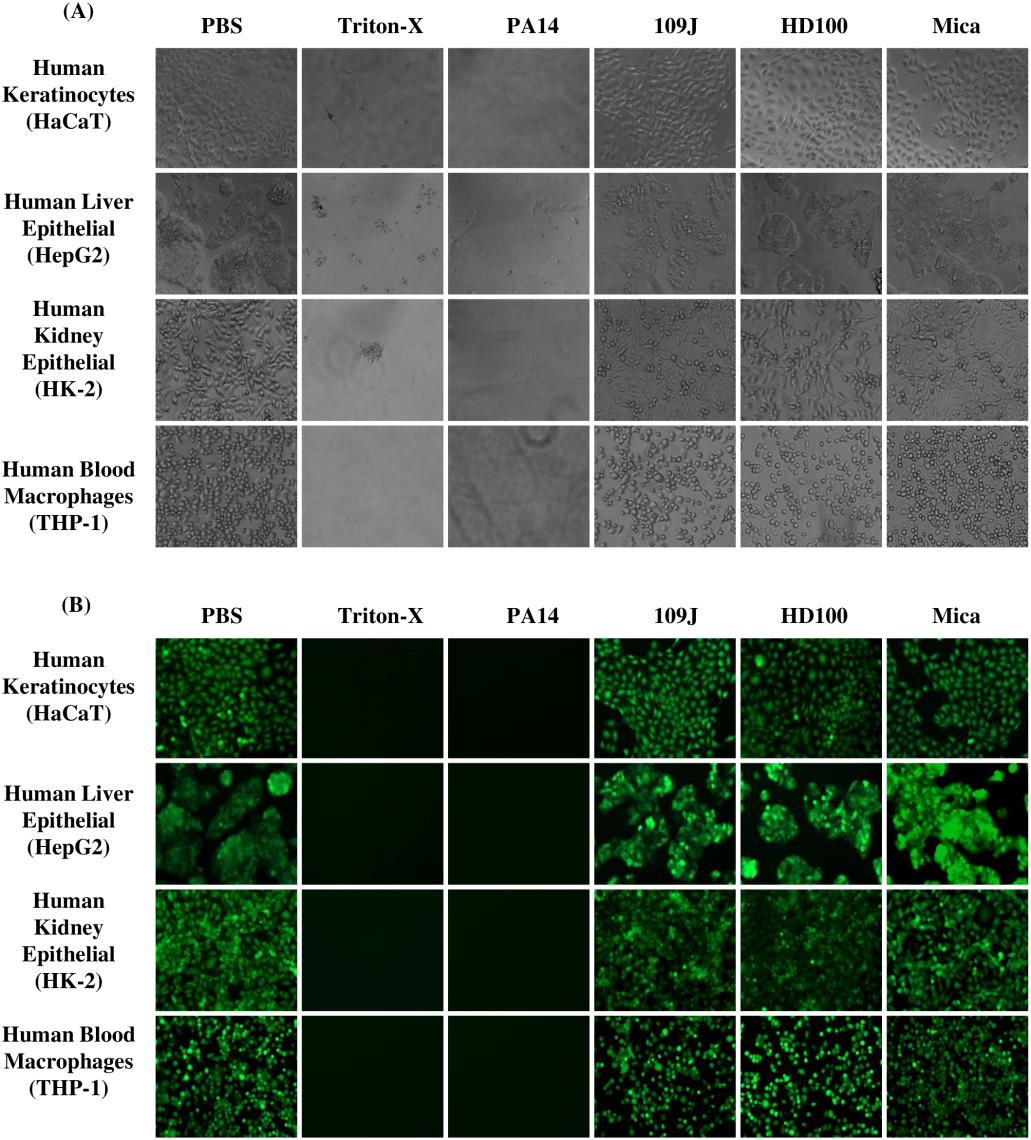
Microscopic imaging of human cell lines following 24 hours exposure to predatory bacteria. Images of HaCaT, HepG2, HK-2, and THP-1 cells 24-hours post-inoculation to *B*. *bacteriovorus* strains 109J, HD100 (~1 x 10^10^ PFU/ml), and *M*. *aeruginosavorus* ARL-13 (~1 x 10^9^ PFU/ml), with PBS as negative control, and Triton X-100 (0.5%) and *P*. *aeruginosa* strain PA14 (~1 x 10^9^ CFU/ml) as positive controls. Cells were washed and stained with Calcein AM Viability Dye. Images were taken using an EVOS FL inverted fluorescence microscope set at 20x magnification (A). Phase contrast images show morphology of human cells (B). Fluorescent images show viable human cells stained green with the viability dye (CaAM).

To validate the cytotoxicity results obtained by PrestoBlue^™^ assay and determine whether there are any visual changes in surface coverage of cells, each cell line was exposed to the predators for 24 hours, washed and examined by light and fluorescent microscopy. As seen in Fig 2A, phase contrast images show no visual changes in the distribution of attached cells in the well in cells exposed to the three predatory bacteria when compared to the cells exposed to PBS control. However, a clear reduction in cell coverage was seen in cells exposed to the positive control Triton-X and the pathogen *P*. *aeruginosa* PA14, demonstrating visible cytotoxic effects on the cells. Additional confirmation was obtained by staining the samples with Calcein AM viability stain. As before, similar cell coverage and cell viability were seen in cells treated by the three predatory bacteria and cells exposed to PBS (Fig 2B).

### Cytokine assays

Since predatory bacteria are Gram-negative bacteria that harbor potentially immunogenic determinants such as lipopolysaccharide (LPS) and flagellum, we were interested to determine whether exposure to predatory bacteria induces an elevated inflammatory response in the human cells. Cell lines were challenged with the three predators and cytokine profiles were analyzed following 4 and 24 hours of exposure (Tables 1 and 2, and S1 and S2 Tables).

**Table 1:** Inflammatory response of five human cell lines to predatory bacteria following 4 hours of exposure.

Cell Lines	Samples	GMCSF	IFN-γ	IL-10	IL-12p70	IL-1β	IL-2	IL-6	IL-8	TNF-α
Human Keratinocytes (**HaCaT**)	***E*. *coli***	**28.45**	-0.63	**2.21**	0.82	-0.02	**6.99**	**3.74**	**2.89**	**28.67**
	***109J***	-0.41	-0.54	0.00	-0.26	0.00	0.00	0.17	-0.19	-0.20
	***HD100***	-0.36	-0.52	0.00	-0.37	0.00	0.00	0.35	-0.13	-0.17
	***Mica***	-0.55	-0.59	0.00	-0.30	0.00	0.00	1.59	-0.08	-0.17
Human Liver Epithelial cells (**HepG2**)	***E*. *coli***	0.51	-0.58	**2.16**	0.56	-0.08	**4.24**	-0.09	**4.65**	-0.17
	***109J***	0.00	0.00	-0.02	0.00	0.00	0.00	0.00	1.33	0.00
	***HD100***	0.00	0.00	0.06	0.00	0.00	0.00	0.00	0.80	0.00
	***Mica***	0.00	0.00	-0.12	0.00	0.00	0.00	0.00	1.16	0.00
Human Kidney Epithelial cells (**HK-2**)	***E*. *coli***	-0.68	-0.17	0.54	-0.09	0.28	**2.28**	-0.83	-0.87	**4.57**
	***109J***	0.22	-0.15	0.00	0.00	0.00	0.00	0.18	0.08	0.60
	***HD100***	-0.01	-0.27	0.00	0.00	0.00	0.00	0.01	-0.15	0.01
	***Mica***	0.10	-0.18	0.00	0.01	0.00	0.00	-0.08	-0.09	-0.05
Human Spleen Monocytes (**MD**)	***E*. *coli***	0.41	-0.11	0.00	0.00	0.00	-0.08	0.21	0.35	1.09
	***109J***	-0.11	-0.24	0.00	0.00	0.00	-0.26	-0.14	-0.10	0.52
	***HD100***	-0.08	-0.44	0.00	0.00	0.00	-0.17	-0.11	-0.11	0.31
	***Mica***	0.16	-0.26	0.00	0.00	0.00	-0.01	0.08	0.12	0.37
Human Blood activated Macrophages (**THP-1**)	***E*. *coli***	**17.11**	-	**6.77**	1.58	**17.63**	-0.14	1.27	0.45	**2.44**
	***109J***	0.67	-	1.97	0.00	**9.36**	0.00	**171.38**	-0.21	1.11
	***HD100***	0.51	-	1.17	0.00	**12.57**	0.00	**191.91**	-0.09	1.43
	***Mica***	**3.48**	-	**3.44**	0.00	**8.61**	0.00	**177.03**	-0.30	0.58

ELISA analysis of GMCSF, IFN- γ, IL-10, IL-12p70, IL-1β, IL-2, IL-6, IL-8 and TNF-α in the human cell lines (HaCaT, HepG2, HK-2, MD, THP-1) exposed to *B*. *bacteriovorus* strains 109J and HD100 (~1 x 10^10^ PFU/ml), and *M*. *aeruginosavorus* ARL-13 (~1 x 10^9^ PFU/ml) for 4 hours. Culture media containing PBS was used as a negative control and *E*. *coli* ATCC 43888 (~1 x 10^8^ CFU/ml) was used as a positive control.

Values represent fold change induction of the cytokines relative to PBS control.

Values in bold represent 2-fold or higher cytokine values relative to PBS control.

Experiments were conducted twice in quadruplicate.

**Table 2:** Inflammatory response of five human cell lines to predatory bacteria following 24 hours of exposure.

Cell Lines	Samples	GMCSF	IFN- γ	IL-10	IL-12p70	IL-1β	IL-2	IL-6	IL-8	TNF-α
Human Keratinocytes (**HaCaT**)	***E*. *coli***	**23.15**	-0.26	**4.05**	0.58	0.82	**10.79**	0.43	1.32	**2.39**
	***109J***	-0.03	-0.39	0.00	-0.21	0.00	0.00	0.30	0.30	-0.50
	***HD100***	0.06	-0.29	0.00	-0.21	0.00	0.00	0.12	0.08	-0.42
	***Mica***	-0.09	-0.36	0.00	-0.28	0.00	0.00	0.13	0.05	-0.60
Human Liver Epithelial cells (**HepG2**)	***E*. *coli***	1.26	-0.58	1.52	0.56	-0.08	**4.60**	-0.09	**13.99**	0.02
	***109J***	0.00	0.00	-0.04	0.00	0.00	0.00	0.00	-0.44	0.00
	***HD100***	0.00	0.00	-0.08	0.00	0.00	0.00	0.00	-0.22	0.00
	***Mica***	0.00	0.00	-0.13	0.00	0.00	0.00	0.00	-0.80	0.00
Human Kidney Epithelial cells (**HK-2**)	***E*. *coli***	-0.74	-0.49	**5.19**	0.14	0.28	**8.53**	-0.17	-0.10	**3.42**
	***109J***	-0.08	-0.03	0.00	-0.07	0.00	0.00	0.00	0.00	-0.11
	***HD100***	-0.03	-0.20	0.00	-0.08	0.00	0.00	0.00	0.00	0.91
	***Mica***	-0.09	-0.14	0.00	-0.04	0.00	0.00	-0.01	0.00	-0.06
Human Spleen Monocytes (**MD**)	***E*. *coli***	0.16	0.02	0.00	0.00	0.00	-0.07	0.05	0.14	0.59
	***109J***	-0.58	0.97	0.00	0.00	0.00	0.02	-0.13	-0.19	0.48
	***HD100***	-0.35	0.57	0.00	0.00	0.00	0.13	-0.15	-0.14	1.29
	***Mica***	-0.30	0.26	0.00	0.00	0.00	-0.10	0.06	-0.03	0.21
Human Blood activated Macrophages(**THP-1**)	***E*. *coli***	**27.59**	-	1.24	1.47	**27.28**	-0.20	0.26	0.78	**10.52**
	***109J***	**203.24**	-	**22.32**	**122.03**	**11.60**	0.00	**82.34**	-0.21	**5.14**
	***HD100***	**187.21**	-	**20.16**	**102.01**	**6.12**	0.00	**78.98**	-0.08	**3.59**
	***Mica***	**278.32**	-	**39.16**	**134.69**	**12.51**	0.00	**78.67**	-0.33	**4.45**

ELISA analysis of GMCSF, IFN- γ, IL-10, IL-12p70, IL-1β, IL-2, IL-6, IL-8 and TNF-α in the human cell lines (HaCaT, HepG2, HK-2, MD, THP-1) exposed to *B*. *bacteriovorus* strains 109J and HD100 (~1 x 10^10^ PFU/ml), and *M*. *aeruginosavorus* ARL-13 (~1 x 10^9^ PFU/ml) for 4 hours. Culture media containing PBS was used as a negative control and *E*. *coli* ATCC 43888 (~1 x 10^8^ CFU/ml) was used as a positive control.

Values represent fold change induction of the cytokines relative to PBS control.

Values in bold represent 2-fold or higher cytokine values relative to PBS control.

Experiments were conducted twice in quadruplicate.

As expected, the *E*. *coli* infected cells showed a greater than 2-fold induction of at least 2 out of the 9 cytokines examined, as compared to PBS control for four out of the five cell lines, with higher fold change increases at 24 hours than at 4 hours of incubation. The predatory bacteria did not cause any significant change in the cytokine levels when compared to the PBS control for four out of the five cell lines tested. For activated blood macrophages, the cells exposed to predatory bacteria showed a notable increase in the cytokines. At 4 and 24 hours of incubation, high levels of IL-1β and IL-6 were measured in cells exposed to *B*. *bacteriovorus*, as compared to PBS controls. In addition, marked increase in levels of GMCSF, IL-10, IL-12p70 and TNF-α was noted after 24 hours of incubation. In cells exposed to *M*. *aeruginosavorus*, more than 2-fold increases in levels of GMCSF, IL-10, and IL-6 was detected at 4 and 24 hours of incubation. In addition, high levels of IL-12p70 and TNF-α were seen after 24 hours of incubation. It was noted that the induction of IL-1β and TNF-α in predator-exposed macrophages was lower than the fold-change increase in values from *E*. *coli* exposed cells.

## Discussion

Widespread and often non-prudent use of conventional antimicrobial agents has led to a drastic surge of multi-drug resistant pathogens [26]. Potential use of predatory bacteria as bio-control agents is gaining momentum, with several recent studies underlining their therapeutic potential [27, 28], specially because of their ability to maintain predation against MDR Gram-negative pathogens regardless of antimicrobial resistance [9].

As with any novel therapeutic agent, it is crucial to determine whether the predatory bacteria are cytotoxic or induce an inflammatory reaction in the host cells. Non-toxic effects of predatory bacteria have been documented in animal models of mice, rabbits, guinea pigs, and chicks [12, 13, 29]. Furthermore, it has been reported that Wild-type isolates of *Bdellovibrio* are unable to proliferate *in vivo* in the absence of a prey, thereby reducing the possibility of prolonged establishment within the mammalian host [30]. Attempts to grow predatory bacteria on mouse liver cells, hamster kidney cells, bovine mammary gland and rabbit ova cells showed no growth of the predators using eukaryotic animal cells as a potential food source, even when predatory bacteria were injected into the rabbit ova cells [31].

*In vivo* animal studies are critical pre-clinical tools in any research and provide invaluable information, but the safety and efficacy from animal studies may not translate into human trials [32, 33]. An initial step in examining the potential effect of a new therapeutic on humans is to first determine its effect on human cells from clinically relevant tissues [34]. In this study, we examined the effect of predatory bacteria on a range of human cells including keratinocytes, liver epithelial cells, kidney epithelial cells, and monocytes from spleen and blood. In this study, two different strains of the periplasmic predator *B*. *bacteriovorus*, which previously showed potential to be used as live antibiotics, as well as one strain of the epibiotic predator *M*. *aeruginosavorus* were used. Regardless of the differences in predatory mechanisms, we found that *B*. *bacteriovorus* and *M*. *aeruginosavorus* did not have any significant deleterious effect on the viability of these human cells, when compared to the viability of PBS control cells (Fig 1). Fluorescent microscopic imaging validated the cytotoxicity assay findings, showing no signs of cell detachment after 24 hours of incubation with predatory bacteria, retaining near-identical morphology to the predator-free cells and exhibiting the integrity of healthy, viable cells (Fig 2). In samples exposed to *P*. *aeruginosa* PA14, no cells were found attached to the surface of the wells. A low level metabolic reading in the viability assay measured with *P*. *aeruginosa* might be the result of single *Pseudomonas* cells adhering to the wells. Although, only a slight increase in growth was measured for *P*. *aeruginosa*, when incubated in cell-free growth medium, it could be suggested that higher *P*. *aeruginosa* growth might have occurred in the experimental human-cell seeded cultures, as *P*. *aeruginosa* could thrive on the expense of the lysed human cells. Thus, *P*. *aeruginosa* CFU numbers at time 24 hour might have been higher than that of the initial inoculation. A slight, but non-significant increase in cell viability after exposure to the predators seen in HaCaT, HepG2 and THP-1 cells, could also be as a result of individual predator cells remaining in the wells, as it was previously demonstrated that the predatory bacteria were able to attach to human corneal epithelial cells (HCLE) [35]. Our data supports earlier findings in which HCLE cells were exposed to high concentrations of *B*. *bacteriovorus* and *M*. *aeruginosavorus*, which were found non toxic to the tested cell line [14].

As predatory bacteria are Gram-negative, their introduction into the human body as “live therapeutics” might give rise to the production of inflammatory cytokines as a response to immunogenic components like LPS [36]. In this study, cytokine levels were measured using HCYTOMAG-60K (Milliplex^®^) immunoassay kit. To maintain consistency, all 9 cytokines were measured, although not all cytokines are produced by all of the tested cell lines. *B*. *bacteriovorus* and *M*. *aeruginosavorus* did not produce a significant elevation of the cytokines in four of the five human cell lines tested (Tables 1 and 2, and S1 and S2 Tables). Our findings conform to the earlier reports of predator exposure on HCLE cells causing no significant induction of the cytokines IL-8 and TNF-alpha [14]. It has been proposed that *B*. *bacteriovorus* may be inherently non-pathogenic to mammalian cells owing to their unique neutral-charged LPS that does not induce a robust immunogenic response in the cell [37]. However, to our knowledge, there are no studies describing the LPS structure of *M*. *aeruginosavorus*. Exposure of blood monocyte-derived and activated macrophages (THP-1) to predatory bacteria caused an elevation of IL-1β and IL-6 at 4 hours, with additional induction of GMCSF, IL-10, IL-12p70 and TNF-α at 24 hours. However, the levels of IL-1β and TNF-α induction from the predator-exposed cells were lower than that from *E*. *coli* exposed cells. While IL-10 has a suppressive effect on pro-inflammatory responses [38, 39], GMCSF is known to induce expression of various cytokines like TNF, IL-1 and other factors, modulating a complex network of immunological responses [40]. The observed induction of various cytokines in the activated blood macrophages is probably a part of the normal endogenous feedback mechanism of the immune system cascade [41]. THP-1 cells are known to express a range of cell receptors that are involved in immunological signaling and their activation with IFN-γ significantly increases the cytokine responses [42]. Additionally, differentiation of THP-1 monocytes to macrophages is known to “prime” the cells for LPS stimulation, resulting in rapid secretion of inflammatory mediators [43, 44] and it might take longer than 24 hours for the inflammatory cytokines to decline. The elevated cytokine levels seen in the predator-exposed wells at 24 hours could be attributed to the higher doses of the predatory bacteria used, as the concentrations of *B*. *bacteriovorus* and *M*. *aeruginosavorus* were two and one log higher, respectively, than that of the *E*. *coli*. Moreover, it might be suggested that during the 24 hours inoculation with *E*. *coli*, some of the of the monocytes might lose viability and stop producing cytokines, whereas cells exposed to the non-virulent predators will remain viable and continue to produce cytokines throughout the 24 hours of incubation, increasing the total cytokine levels accumulating in the well.

Our work is in accord with *in vivo* studies done with chicks and mice that highlight the non-toxic effects of these predators. It was demonstrated that oral administration of *B*. *bacteriovorus* was able to control *Salmonella* infection in young live chicks, significantly reducing cecal inflammation, without causing any adverse effect on the growth and well being of the chicks [12]. It was recently shown that *B*. *bacteriovorus* and *M*. *aeruginosavorus* were safe and non-immunogenic in mice when injected via intranasal and intravenous route [13]. The study also demonstrated that the predatory bacteria did not provoke a sustained elevation of any of the inflammatory cytokines and were cleared efficiently from the tissues.

In conclusion, this study expands upon our previous knowledge and highlights the non-pathogenic attributes of *B*. *bacteriovorus* and *M*. *aeruginosavorus* on a diverse range of human cells. *In vitro* studies on human cells are an important addition to animal studies to provide a better estimate of the successful translation of animal models to human clinical trials. This study demonstrated that a direct exposure of predatory bacteria to different cell lines did not elicit any measurable cytotoxic or inflammatory response. Establishing that these predatory bacteria are non-toxic to humans, in addition to their ability to control MDR bacteria and biofilms, make them attractive alternatives or adjuncts to antibiotics. This study is a step forward in paving the way for future clinical trials using predatory bacteria as safe biological control agents against human pathogens.

## Supporting Information

S1 Table Inflammatory response of five human cell lines to predatory bacteria following 4 hours of exposure. ELISA analysis of GMCSF, IFN- γ, IL-10, IL-12p70, IL-1β, IL-2, IL-6, IL-8 and TNF-α in the human cell lines (HaCaT, HepG2, HK-2, MD, THP-1) exposed to *B*. *bacteriovorus* strains 109J and HD100 (~1 x 10^10^ PFU/ml), and *M*. *aeruginosavorus* ARL-13 (~1 x 10^9^ PFU/ml) for 4 hours. Culture media containing PBS was used as a negative control and *E*. *coli* ATCC 43888 (~1 x 10^8^ CFU/ml) was used as a positive control. Values represent average concentration of cytokine levels (pg/ml) ± standard deviation. Experiments were conducted twice in quadruplicate. Values in bold represent 2-fold or higher cytokine values relative to PBS control.
(TIF)

S2 Table Inflammatory response of five human cell lines to predatory bacteria following 24 hours of exposure. ELISA analysis of GMCSF, IFN- γ, IL-10, IL-12p70, IL-1β, IL-2, IL-6, IL-8 and TNF-α in the human cell lines (HaCaT, HepG2, HK-2, MD, THP-1) exposed to *B*. *bacteriovorus* strains 109J and HD100 (~1 x 10^10^ PFU/ml), and *M*. *aeruginosavorus* ARL-13 (~1 x 10^9^ PFU/ml) for 24 hours. Culture media containing PBS was used as a negative control and *E*. *coli* ATCC 43888 (~1 x 10^8^ CFU/ml) was used as a positive control. Values represent average concentration of cytokine levels (pg/ml) ± standard deviation. Experiments were conducted twice in quadruplicate. Values in bold represent 2-fold or higher cytokine values relative to PBS control.
(TIF)
