# Supplementary figures and images for: Effect of Predatory Bacteria on Human Cell Lines
Source: PLoS One. 2016 Aug 31;11(8):e0161242. doi: 10.1371/journal.pone.0161242 (PMC5006992; doi:10.1371/journal.pone.0161242)

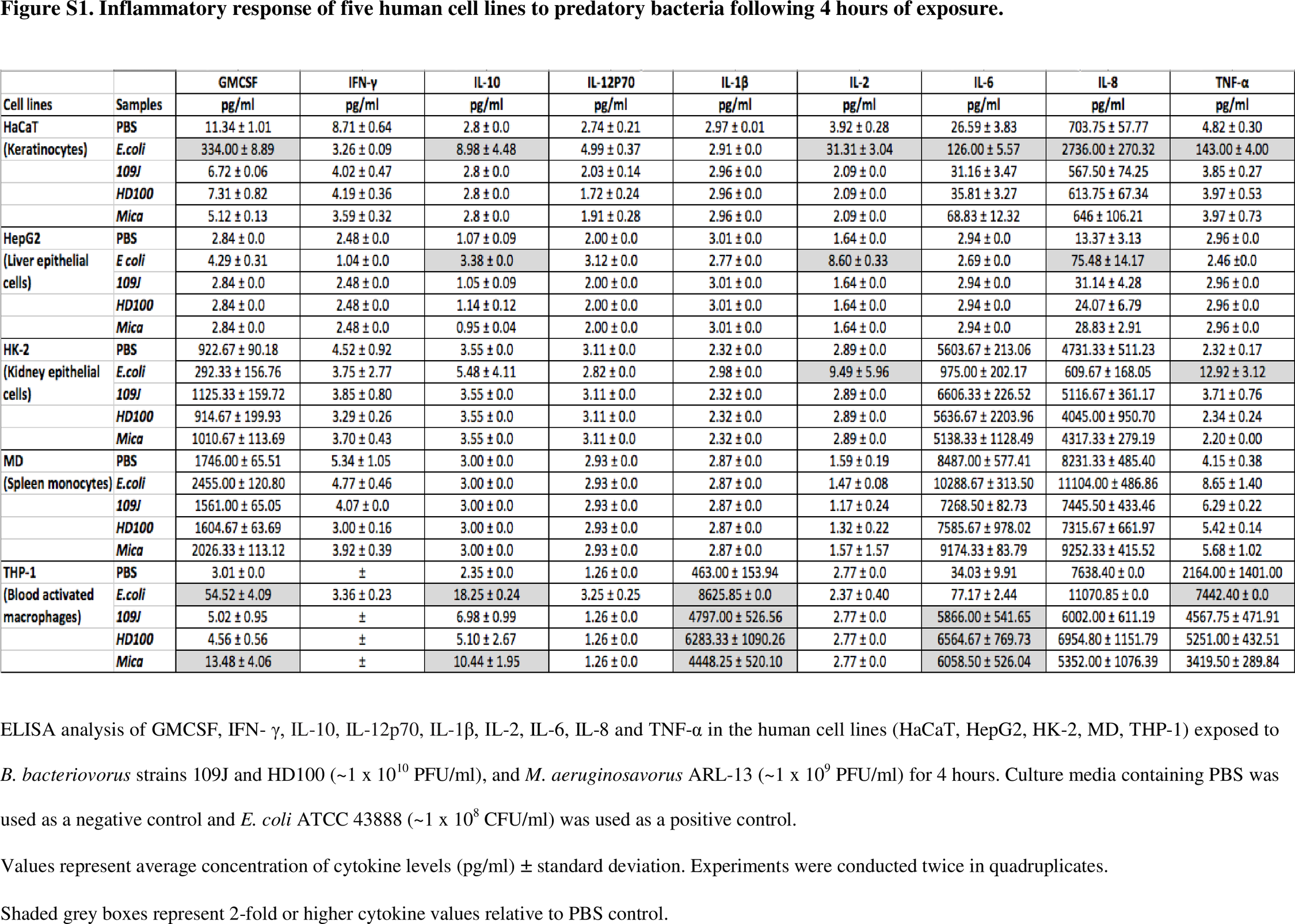

Supplement: S1 Table — ELISA analysis of GMCSF, IFN- γ, IL-10, IL-12p70, IL-1β, IL-2, IL-6, IL-8 and TNF-α in the human cell lines (HaCaT, HepG2, HK-2, MD, THP-1) exposed to B. bacteriovorus strains 109J and HD100 (~1 x 10^10^ PFU/ml), and M. aeruginosavorus ARL-13 (~1 x 10^9^ PFU/ml) for 4 hours. Culture media containing PBS was used as a negative control and E. coli ATCC 43888 (~1 x 10^8^ CFU/ml) was used as a positive control. Values represent average concentration of cytokine levels (pg/ml) ± standard deviation. Experiments were conducted twice in quadruplicate. Values in bold represent 2-fold or higher cytokine values relative to PBS control. (TIF) [file pone.0161242.s001.tif]

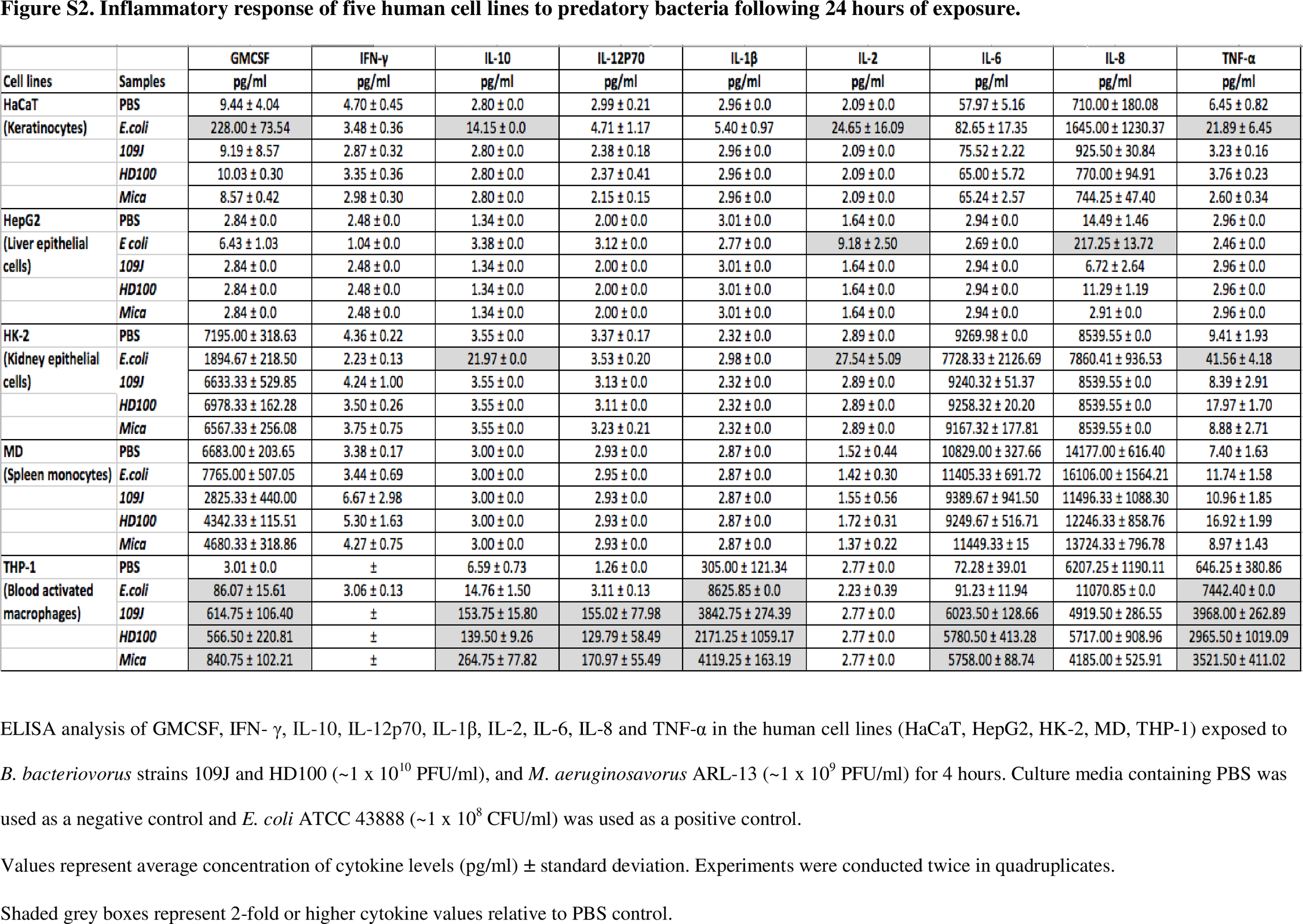

Supplement: S2 Table — ELISA analysis of GMCSF, IFN- γ, IL-10, IL-12p70, IL-1β, IL-2, IL-6, IL-8 and TNF-α in the human cell lines (HaCaT, HepG2, HK-2, MD, THP-1) exposed to B. bacteriovorus strains 109J and HD100 (~1 x 10^10^ PFU/ml), and M. aeruginosavorus ARL-13 (~1 x 10^9^ PFU/ml) for 24 hours. Culture media containing PBS was used as a negative control and E. coli ATCC 43888 (~1 x 10^8^ CFU/ml) was used as a positive control. Values represent average concentration of cytokine levels (pg/ml) ± standard deviation. Experiments were conducted twice in quadruplicate. Values in bold represent 2-fold or higher cytokine values relative to PBS control. (TIF) [file pone.0161242.s002.tif]
